# Generalizing remotely supervised transcranial direct current stimulation (tDCS): feasibility and benefit in Parkinson’s disease

**DOI:** 10.1186/s12984-018-0457-9

**Published:** 2018-12-07

**Authors:** Bryan Dobbs, Natalie Pawlak, Milton Biagioni, Shashank Agarwal, Michael Shaw, Giuseppina Pilloni, Marom Bikson, Abhishek Datta, Leigh Charvet

**Affiliations:** 1 New York University Langone Health, New York, USA; 2 Tufts School of Medicine, Boston, USA; 3 City College of New York, New York, USA; 4 Soterix Medical, New York, USA; 5 NYU Comprehensive MS Care Center, 240 East 38th Street, 20th Floor, New York, NY 10016 USA

**Keywords:** Transcranial direct current stimulation, tDCS, Telerehabilitation, Parkinson’s disease, Multiple sclerosis

## Abstract

**Background:**

Transcranial direct current stimulation (tDCS) is a non-invasive brain stimulation technique that has been shown to improve common symptoms of neurological disorders like depressed mood, fatigue, motor deficits and cognitive dysfunction. tDCS requires daily treatment sessions in order to be effective. We developed a remotely supervised tDCS (RS-tDCS) protocol for participants with multiple sclerosis (MS) to increase accessibility of tDCS, reducing clinician, patient, and caregiver burden. The goal of this protocol is to facilitate home use for larger trials with extended treatment periods. In this study we determine the generalizability of RS-tDCS paired with cognitive training (CT) by testing its feasibility in participants with Parkinson’s disease (PD).

**Methods:**

Following the methods in our MS protocol development, we enrolled sixteen participants (*n* = 12 male, *n* = 4 female; mean age 66 years) with PD to complete ten open-label sessions of RS-tDCS paired with CT (2.0 mA × 20 min) at home under the remote supervision of a trained study technician. Tolerability data were collected before, during, and after each individual session. Baseline and follow-up measures included symptom inventories (fatigue and sleep) and cognitive assessments.

**Results:**

RS-tDCS was feasible and tolerable for patients with PD, with at-home access leading to high protocol compliance. Side effects were mostly limited to mild sensations of transient itching and burning under the electrode sites. Similar to prior finding sin MS, we found preliminary efficacy for improvement of fatigue and cognitive processing speed in PD.

**Conclusions:**

RS-tDCS paired with CT is feasible for participants with PD to receive at home treatment. Signals of benefit for reduced fatigue and improved cognitive processing speed are consistent across the PD and MS samples. RS-tDCS can be generalized to provide tDCS to a range of patients with neurologic disorders for at-home rehabilitation.

**Trial registration:**

ClinicalTrials.gov Identifier: NCT02746705. Registered April 21st 2016.

## Background

Transcranial direct current stimulation (tDCS) is a noninvasive neuromodulation technique that uses surface electrodes placed on the scalp to deliver a low amperage (typically 1.5–2.0 mA) direct current to a targeted cortical region. The mechanism of tDCS, based on current scientific consensus, is that the direct current enhances neural plasticity and enriches any rehabilitative training or learning completed during the treatment session [1, 2]. Each session of tDCS typically lasts 20 min while the user typically completes simultaneous therapy or training. Greater and more persistent benefit has been observed following multiple stimulation sessions, suggesting a cumulative and long term treatment for maximal benefit. tDCS has been shown to be a safe technique often accompanied by only mild and transient adverse events like skin tingling [3, 4].

In order to reach participants for long-term study, treatments must be delivered at home. Requiring participants to attend clinic every weekday for treatment is not feasible due to professional and personal obligations alongside any disability they may be managing. Many studies to date in tDCS have been underpowered and are limited to small sample sizes with few sessions studied (i.e., 10 sessions or less) [3].

We developed a remotely supervised tDCS protocol (RS-tDCS) where participants are able to complete daily 20-min sessions from home while supervised by a study technician using real-time monitoring via videoconference. We have previously verified the feasibility and tolerability of this protocol in a cohort of patients with multiple sclerosis (MS) [4–7]. Our RS-tDCS protocol allows for rapid recruitment and extended study schedules, expanding our sample size and allowing participants to complete 20 tDCS sessions or more, exceeding most studies in tDCS. The RS-tDCS protocol enables easy study of the long-term effects of tDCS in MS and has found significant benefits for mood, fatigue, and cognitive impairment [8, 9].

Here we expand our RS-tDCS protocol to people with Parkinson’s disease (PD). PD is a chronic, degenerative neurological disorder that can produce a range of motor and non-motor disability [10]. Many of the pharmacological and surgical therapies are targeted towards improving motor symptoms. Non-motor symptoms such as sleep disturbances, cognitive impairment, depression and fatigue remain a major cause of disability that can lead to overall deteriorations in quality of life [11, 12]. Recent reports have demonstrated that cognition, such as executive functioning and visuospatial processing, are positively associated with quality of life in PD patients [13]. For this reason there is growing interest in managing and treating the neuropsychiatric symptoms that occur with the disorder. Neurostimulation techniques such as deep brain stimulation (DBS), transcranial magnetic stimulation (TMS) and tDCS have shown to be effective for ameliorating motor symptoms [14–16] but the efficacy of these modalities for non-motor symptoms is still being studied [17, 18]. While DBS focuses on deep brain structures that are not thought to be modulated by transcranial neurostimulation, TMS has similar targets to tDCS with both focusing on cortical areas. TMS and tDCS may have similar benefits [19, 20], but TMS comes with higher costs and no option for home-based, remotely supervised sessions. Furthermore, the combination of tDCS with traditional therapeutic treatment has been used recently to enhance improvement of motor and non-motor symptoms in neurological diseases. In the literature, several studies demonstrated positive effects on motor and cognitive impairments in PD patients after multiple sessions of physical or cognitive training combined simultaneously with tDCS [21–23]. In particular, studies have documented the beneficial effect of anodal tDCS over the left dorsolateral prefrontal cortex (DLPFC), alone and in combination with computerized cognitive training, on both mood disturbances and cognitive performance (language, attention and executive functions) [22, 24, 25].

We planned to expand this RS-tDCS therapy protocol to PD participants, predicting that participants with PD would tolerate RS-tDCS in a similar to those with MS. We recruited participants with PD in an open-label RS-tDCS study following the methods of original study in MS [6, 7]. Our findings include feasibility, tolerability, and preliminary efficacy of tDCS in people with PD.

## Methods

Participants with a confirmed diagnosis of PD were recruited into this open-label feasibility study. All study procedures were approved by the New York University School of Medicine Institutional Review Board. Written, informed consent was obtained from each participant.

Study eligibility criteria were purposefully broad to assess the feasibility of our RS-tDCS protocol in a Parkinson’s cohort. The criteria required that patients had a definite diagnosis of PD, were between the ages of 30–89, had no history of serious brain trauma, and were physically, visually, and cognitively competent enough to perform study procedures. Furthermore, participants were required to have adequate facilites at home to carry out the telerehabilitation protocol. Participants unable to physically perform study procedures were required to enroll with a proxy.

### Equipment

**Fig. 1:**
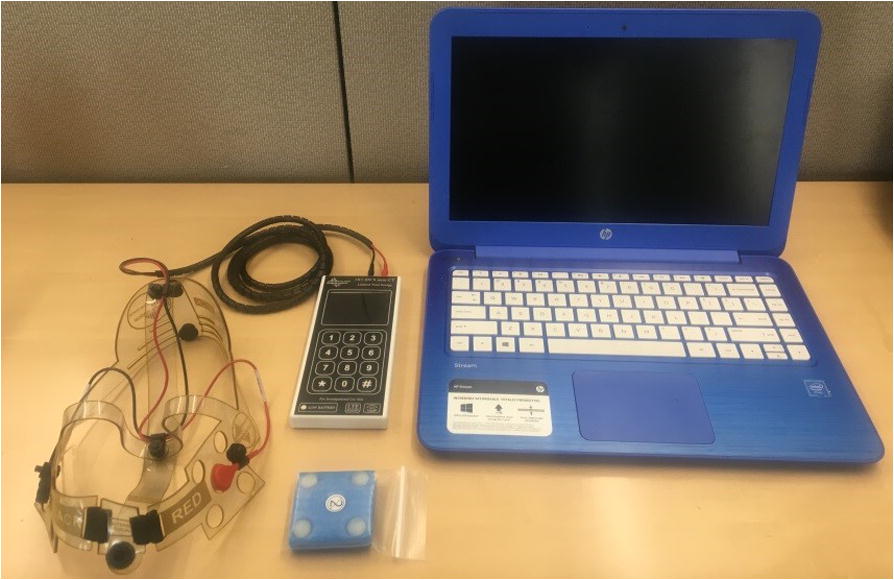
Study Equipment

**Fig. 2:**
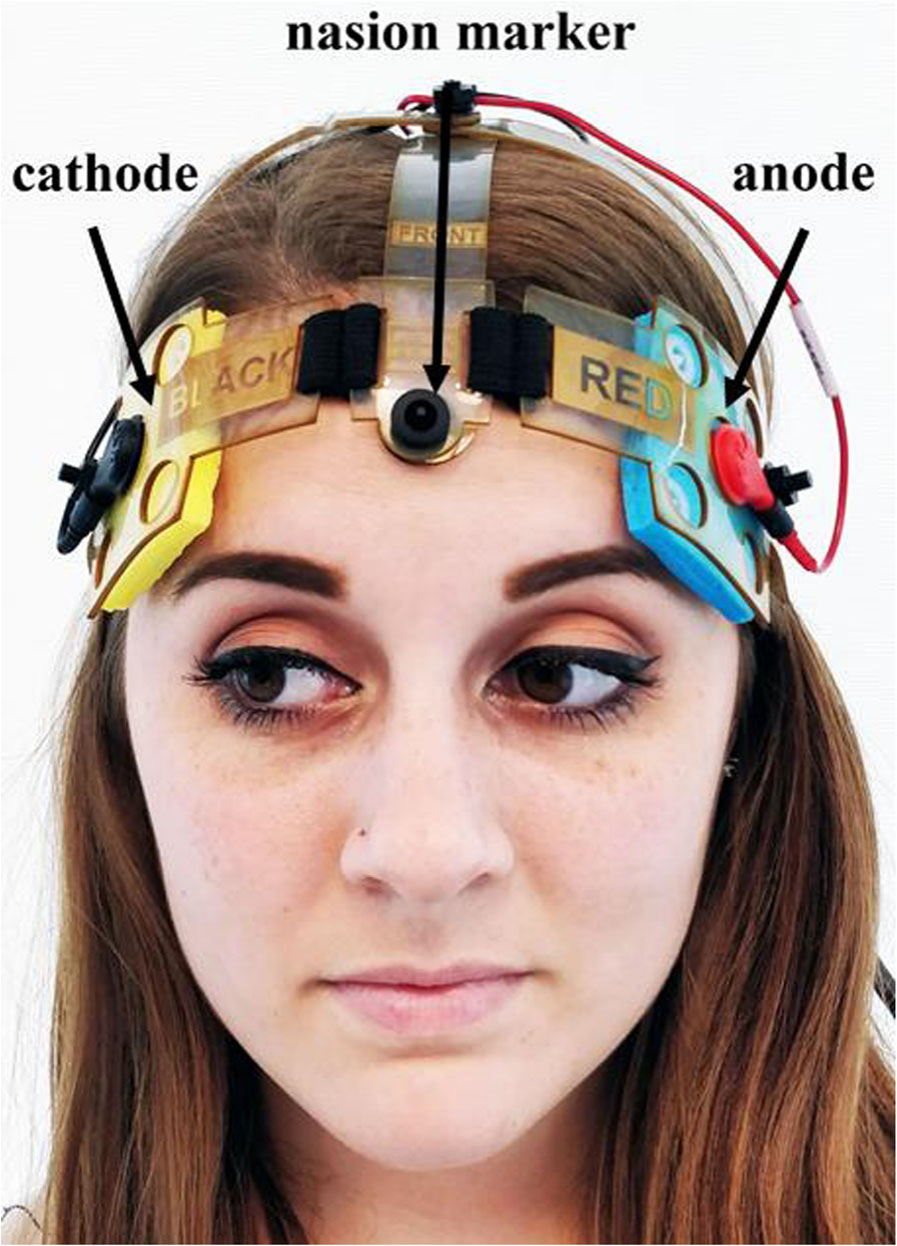
Easystrap: nasion head strap used for electrode placement

Participants were given a Soterix Mini-CT [11] device to use for the duration of the study. The Mini-CT is a small, rechargeable battery powered unit that has been designed for safety and ease of use, see Fig. 1. The device samples impedance and current output alongside session data to ensure standardized and high-quality stimulation across all sessions. The stimulation current is delivered by the Soterix EasyStrap [11] using DLPFC montage with electrodes resting at F3 and F4 according to the 10–20 EEG system (left anodal). Specifically, the EasyStrap allows for consistent placement targeting with the anode placed over left DLPC and the cathode over the right supraorbital area. The correct position of the nasion head strap could be assessed by the technician using two visual markers, placed one in the front (in line with the nose) and one in the back (in line with the inion) as shown in Fig. 2. An individually packaged, pre-moistened sponge electrode (5 cm × 5 cm) was used for each session. Participants were also given a laptop computer (HP Stream, 15′) to perform daily study procedures and connect to study technicians.

**Fig. 3:**
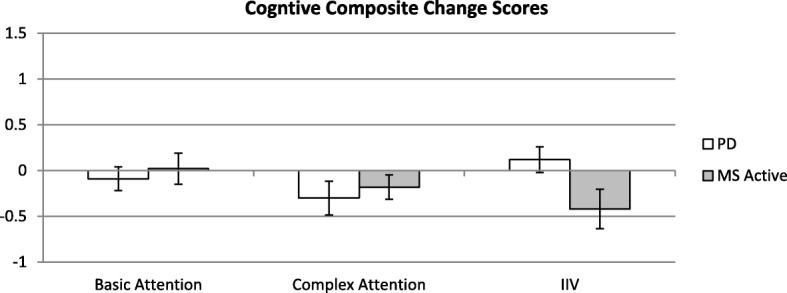
Bars depict mean change in composite score with error bars representing standard error of the mean. Negative values indicate improvement

**Fig. 4:**
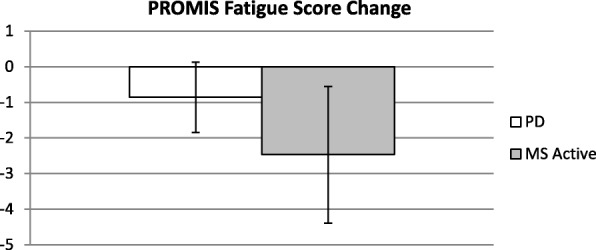
Bars depict mean change in PROMIS Fatigue scale with error bars representing standard error of the mean. Negative values indicate improvement

### RS-tDCS protocol

The RS-tDCS protocol consists of a baseline study visit performed in clinic, daily tDCS sessions completed at home, and a follow-up visit performed in clinic.

At baseline, participants were consented and screened for eligibility criteria. Participants completed questionnaires and neuropsychological testing under the supervision of a study technician. Finally, the study technician trained the participants (and, if needed, the healthcare proxy) on use of the tDCS device and the daily protocol for RS-tDCS sessions. Training included a tolerability test and instructional video that guided operation of study equipment. Following training, study technician confirmed that the participant was competent and able to replicate the study procedures from home.

Participants completed a total of ten × 20 min tDCS sessions (2.0 mA, DLPFC montage, left anodal) paired with cognitive training (CT). Each daily session, as well as baseline and follow up visit, was scheduled at the same time every day within three hours of the participant taking L-Dopa medication.

Each at-home session typically required a total of 30 min. The study technician connected to the participant’s laptop via remote desktop software (TeamViewer [26]) and initiated the video call using HIPAA compliant video conferencing software (VSee [27]). The participant attached the pre-moistened sponge electrodes to the headset via snap buttons and placed it on their head. The study technician then confirmed proper placement of the headset. The participant turned on the device and visually confirmed (holds the device up so that the technician can see) contact quality. Once ‘Good’ or ‘Moderate’ contact was achieved the technician gave the participant the three-digit dose code to unlock the device for the 20-min session. During the 20-min stimulation period, participants played a pre-selected assortment of cognitive training games targeting processing speed and working memory (Lumos Labs [28]).

### Study measures

#### Baseline/follow-up measures

Participants were required to have a reading recognition ability as measured by the the Wide Range Achievement Test 3 (WRAT-3) standard score at least in the average range (i.e., 85 or above. The reading recognition score serves as a proxy of premorbid IQ [29] and ensures adequate understanding of study instructions.

Cognitive assessments consisted of both computerized and pen-and-paper testing. Participants completed the Symbol Digits Modalities Test (SDMT) [30], Rey Auditory Verbal Learning Test (RAVLT) [31], and Brief Visuospatial Memory Test (BVMT-R) [32]. Participants also completed the computer-based Cogstate Brief Battery [33] (measuring simple reaction time, choice reaction time, and n-back tasks) and Attention Network Test - Interaction [34] (measuring orienting, alerting and executive attention networks as well as intra-individual variability in reaction time). Both computer-based measures provide a more sensitive and extended assessment of cognitive processing speed and efficiency that have previously responded to tDCS treatment [8]. Self-Reported Measures included Patient Reported Outcomes Measurement Information System (PROMIS) [35] forms focused on quality of life (fatigue, pain, affect, and sleep). Participants also completed the Positive and Negative Affect Schedule (PANAS) [36] to measure treatment effects on affect and mood.

The Unified Parkinson’s Disease Rating Scale (UPDRS) [37] was administered at baseline and follow-up by a clinician specializing in PD.

#### Daily measures

Each day study technicians asked participants about sensations or adverse events related to the tDCS stimulation as well as symptom specific measures.

Before, during, and after each session participants were asked to report any pain they experienced from the tDCS stimulation on a 0–10 visual analog scale (easily accessible at all times). Technicians asked patients to further elaborate on specific intensities of such sensations (ie. burning sensations, tingling sensations, etc.) they felt during or in between tDCS sessions.

Participants were also asked to rate any fatigue they might have on a 0–10 visual analogue scale and complete the PANAS questionnaire before and after each session.

### Analyses

Analysis was completed using IBM SPSS Statistics 23 software. Analysis focused on descriptive statistics of adverse events and sensations due to tDCS. Preliminary efficacy analysis focused on Cohen’s *d* effect size but also included paired-sample t-tests to test for any significant within-subject differences from baseline to follow-up.

## Results

A total of *n* = 16 participants with PD were enrolled with demographic and clinical features shown in Table 1, and only one of them was assisted by caregiver. All the enrolled participants had a WRAT-3 score of 85 or above, as requested by the inclusion criteria (Table 1).

**Table 1:** Demographic Characteristics (*n* = 16)

Demographic characteristics	Values
Age (Mean ± SD)	66.9 ± 5.4
Gender (% Male)	80%
Race (% White)	100%
Years Education (Mean ± SD)	17.5 ± 2.6
Baseline WRAT-3 (Mean ± SD)	117.5 ± 12.5
Baseline UPDRS Total (Mean ± SD)	39.6 ± 12.0

### Compliance

All but one participant (*n* = 15) completed all ten RS-tDCS for full compliance. One participant was discontinued from treatment after two study sessions due to a medical issue (cardiac event) judged to be unrelated to the stimulation.

### Adverse events

Adverse events are shown below in Table 2. Adverse events were mostly restricted to sensations of tingling and burning on the electrode site. There were few occurrences of headache or head pain, and one occurrence of ‘difficulty concentrating’. All reported adverse events were commonly reported side effects of tDCS and were mild. All of these events resolved after completion of the stimulation session.

**Table 2:** Adverse Events Experienced over 152 tDCS Sessions (*n* = 16, full sample)

Adverse Event	Tingling	Burning	Localized Head Pain	Itching	Headache	Difficulty Concentrating
Frequency (%)	43.42	28.95	7.89	7.89	5.92	0.66
Average Intensity	2.24	2.36	2.88	2.58	2.72	1.00
Average Duration	11.70	8.31	10.08	2.71	8.50	1.00

Intensity of adverse events was rated on a 1–10 scale

The max adverse event duration was 20 min, or the entire length of the session

### Preliminary efficacy

We tested tDCS response across multiple symptom domains to determine preliminary efficacy of tDCS in a PD sample and then compared these results to our findings in MS [33, 34]. Not all participants were able to complete all outcome measures due to disease disability and later inclusion of assessment measures. Table 3, below, details these data.

**Table 3:** Preliminary efficacy

	Baseline (Mean ± SD)	Follow-Up (Mean ± SD)	Cohen’s *d*	*p* - value	n
Visual Learning – BVMT-R	16.3 ± 6.6	20.3 ± 5.8	0.64	0.50	13
Reaction Time – Cogstate	2.55 ± 0.04	2.53 ± 0.06	− 0.46	0.19	8
Choice Reaction Time – Cogstate	2.75 ± 0.06	2.72 ± 0.07	−0.38	0.08	7
Negative Affect - PANAS	16.4 ± 5.0	13.8 ± 3.5	−0.59	0.01*	11
Positive Affect - PANAS	36.4 ± 7.3	34.3 ± 7.5	−0.19	0.24	12
PROMIS Fatigue	18.6 ± 3.5	16.9 ± 3.8	−0.45	0.10	12
PROMIS Sleep	31.4 ± 5.3	29.1 ± 6.3	−0.40	0.16	12

*Statistical significance at *p* > 0.05 level. Not all participants had the capacity to complete all assessments due to motor impairment

Brief Visuospatial Memory Test Revised (BVMT-R) [32]

Cogstate Brief Battery [33]

Positive and Negative Affect Schedule (PANAS) [36]

Patient Reported Outcomes Measurement Information System (PROMIS) [35]

### Comparison to findings from RS-tDCS in MS patients

We have previously reported data in MS utilizing our RS-tDCS protocol showing benefits for cognition and fatigue [33, 34]. Below, we compared results from this PD cohort to results from a feasibility study with the similar treatment methodology in MS (the only difference being the MS cohort had 1.5 mA stimulation vs. 2.0 mA in the PD cohort). The MS sample consisted of 39 participants: *n* = 19 participants were in an open-label, active RS-tDCS study with the same protocol as described above (MS Active).

We continued to compare tolerability and side effect data of the PD group and MS active group. No data is presented from the MS control group as they had no tDCS device or stimulation involved in their daily treatments. Table 4, below, display these data.

**Table 4:** RS-tDCS Tolerability in Comparable in PD and MS Samples

	PD		MS	
	Frequency	Avg. Intensity (1–10)	Frequency	Avg. Intensity (1–10)
Skin Tingling	43%	2.2	64%	2.5
Skin Itching	8%	2.6	26%	2
Burning Sensation	29%	2.4	26%	3.1
Nausea	0%	0	4%	3.0
Headache	6%	2.7	3%	1.8
Facial Muscle Twitching	0%	0	0%	0
Blurred Vision	0%	0	1%	1
Localized Head Pain or Pressure	8%	2.9	4%	2.6
Forgetfulness	0%	0	1%	4.5
Difficulty Concentrating	1%	1	1%	2.3
Dizziness	0%	0	1%	2.5
Difficulty Breathing	0%	0	0%	0

Figure 3, below, shows results from our computerized cognitive battery. We developed three composites for basic attention (ANT-I Orienting and Attention Networks, Cogstate Detection speed), complex attention (ANT-I Executive Network, Cogstate Identification and One-Back speeds), and response variability (intra-individual variability (IIV) of ANT-I and Cogstate Identification). Scores from Cogstate and the ANT-I were converted to z-scores using the entire data set (PD, MS active, MS control) in order to give each component of the composites equal weight.

Figure 4, below, depicts change in fatigue compared between the active stimulation groups as determined by change in the PROMIS fatigue scale.

## Discussion

RS-tDCS is a feasible and tolerable method to provide in-home treatment for PD with full protocol compliance (100%). We have previously reported data related to the feasibility and efficacy of our RS-tDCS protocol for people with MS. The tolerability and feasibility of RS-tDCS in two cohorts of people with neurologic disorders suggests the generalizability of our RS-tDCS protocol to many more patient cohorts.

Promising treatment effects were found for fatigue, mood, and sleep improvement alongside gains in cognitive processing speed and visual learning. These findings suggest that DLPFC stimulation can improve quality of life across symptomatic domains for people with PD.

Comparisons to the previous findings in MS indicate that our RS-tDCS protocol had a similar tolerability profile and benefits in people with PD [8, 9]. We also see a similar benefit in complex attention in both cohorts, but slight differences in fatigue and IIV benefit. It is possible that these differences are a result from demographic differences in the cohorts (the PD group was older, more homogeneous, and had higher levels of estimated premorbid intellectual functioning).

Our PD sample had a higher proportion of white male participants with more education than the larger PD population. A greater proportion of white males is somewhat common among PD study cohorts [38], and we do not believe that this represents a major confound to our study goals. The primary purpose of this work is to expand the remotely supervised procedures to those with PD, and we were successful in demonstrating that this protocol is feasible in this group. In our prior studies in MS, where women are actually represented more than men, we have not found that gender or education has influenced the success of the protocol. Instead, we are encouraged that even those patients with severe levels of disability (e.g., wheelchair dependent and limited use of hands) or with cognitive impairment (e.g., secondary to MS) have been able to successfully participate. This study serves to expand these findings beyond MS and into a new patient population, PD, which may also have therapeutic benefit from the use of tDCS. However, future studies in PD should be careful to include as diverse a range of participants as possible in order to be sure that findings are applicable to the full PD population.

These findings support further use of the RS-tDCS protocol for people with PD. While the DLPFC electrode montage can provide quality of life improvements, it is also worth investigating if a different combination of montages and paired trainings can alleviate specific PD problems and symptoms.

## Conclusions

RS-tDCS is feasible for people with PD and preliminary efficacy indicates symptomatic benefit and generalizable across a range of neurological conditions.
